# Endovascular reconstruction of aortic bifurcation for aortic pseudoaneurysm in a pediatric trauma patient

**DOI:** 10.1016/j.jvscit.2023.101140

**Published:** 2023-03-05

**Authors:** K. Benjamin Lee, Antonio Solano, M. Shadman Baig, Gerardo Gonzalez-Guardiola, Carlos H. Timaran, Melissa R. Keller, Melissa L. Kirkwood, Michael Shih

**Affiliations:** Division of Vascular and Endovascular Surgery, Department of Surgery, University of Texas Southwestern Medical Center, Dallas, TX

**Keywords:** Aorta, Endovascular therapies, Pediatric vascular surgery, Pseudoaneurysm, Trauma

## Abstract

Endovascular treatment options for vascular injury in pediatric patients are quite limited owing to concerns regarding long-term durability and the lack of devices suitable for the pediatric anatomy. However, in rare circumstances, open surgical therapy will not be an option, and patients will require unconventional endovascular solutions for lifesaving or limb-saving therapies. In the present report, we describe an endovascular treatment of a pediatric patient for whom initial surgical management of a blunt abdominal aortic injury had failed, with subsequent development of an aortic pseudoaneurysm. A 10-year-old girl had presented after a high-speed motor vehicle accident with a seatbelt sign. Multiple abdominal injuries were identified, including blunt aortic injury, significant devitalization of the small bowel, colonic perforation with fecal contamination, multiple lumbar spine fractures, and pulmonary contusions. The patient developed bilateral lower extremity ischemia from the aortic injury and had initially undergone open repair. One month later, the patient had developed a pseudoaneurysm of the aorta near the aortic bifurcation. Because of the hostile abdomen and ensuing short gut syndrome, the pseudoaneurysm was managed using endovascular techniques. The limb of an Excluder internal iliac branch endoprosthesis (W.L. Gore & Associates, Flagstaff, AZ) was used as the endograft. The aortic bifurcation was raised and reconstructed using four Viabahn self-expanding stents (W.L. Gore & Associates). The completion angiogram showed complete resolution of the pseudoaneurysm. The follow-up computed tomography angiogram showed widely patent stent grafts with complete resolution of the pseudoaneurysm. Endovascular management of traumatic vascular injuries in pediatric patients is feasible. The likelihood of reintervention in the future is high with patient growth. However, it is a viable option in lifesaving or limb-saving situations in which open repair is high risk.

Trauma is a leading cause of morbidity and mortality in childhood, and blunt trauma accounts for 80% to 90% of abdominal injuries. Blunt abdominal aortic trauma (BAAT) is uncommon in pediatric patients, but this type of injury is frequently fatal.^1^, ^2^, ^3^ Traumatic vascular injuries in pediatric patients will almost universally be managed with open surgical techniques because no endovascular devices have been approved for use in pediatric patients. However, in rare circumstances, the risk of open surgical therapy will outweigh the potential benefits. In the present report, we have described a case of endovascular reconstruction of the aortic bifurcation for early degeneration and pseudoaneurysm formation after open repair of BAAT in a pediatric patient.

## Case report

**Fig 1:**
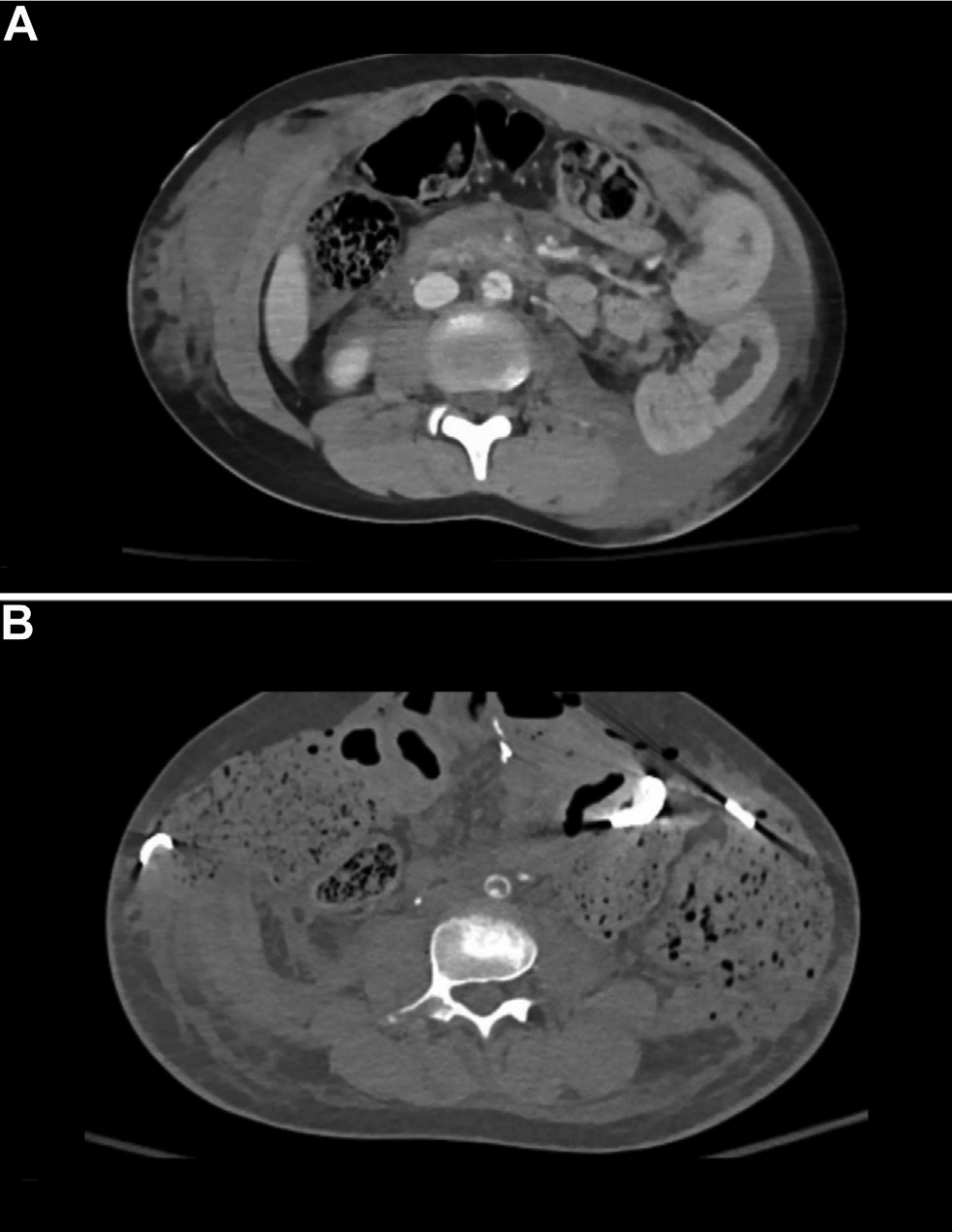
**A,** Initial computed tomography (CT) angiogram after the injury showing focal aortic dissection. **B,** Subsequent CT angiogram showing evolution of the aortic injury to worsening dissection and near occlusion of the aorta.

A 10-year-old girl had presented to the emergency room of a level 1 pediatric trauma center after a high-speed motor vehicle collision. Primary and secondary examinations revealed a prominent seatbelt sign, tachycardia, left neck abrasion, and peritonitis. Her extremities had palpable pulses and no signs of motor or sensory deficits. A computed tomography (CT) scan revealed multiple intra-abdominal injuries, including BAAT with focal dissection, a traumatic abdominal wall hernia, free fluid in the abdomen, and L2-L3 spine fractures (Fig 1, *A*). The patient was taken to the operating room for damage control laparotomy and was found to have fecal peritonitis with multiple segments of devitalized small and large bowel. The patient underwent multiple small bowel resections (>50% of small bowel resected), ileocecectomy, descending colectomy, and abdominal closure. The BAAT was initially managed conservatively. However, 12 hours after the initial injury, the patient had lost her pedal pulses. A repeat CT angiogram showed progression of the aortic injury resulting in occlusion of the distal abdominal aorta (Fig 1, *B*). The patient was taken to the operating room for emergent abdominal exploration. In the operating room, the infrarenal abdominal aorta was clamped and aortotomy performed. Significant intraluminal thrombus secondary to dissected intimal flap was present, and an intramural hematoma was observed. Debridement of the injured aorta and thromboendarterectomy were performed. Because of the extensive fecal contamination in the abdomen from the initial injury, a decision was made not to use any prosthetic material. Instead, a segment of the great saphenous vein was harvested, and patch angioplasty of the aorta was performed. Palpable pedal pulses had been restored at the end of the case. Subsequently, the patient was returned to the operating room multiple times for serial washouts, reestablishment of bowel continuity, and abdominal wall reconstruction.

**Fig 2:**
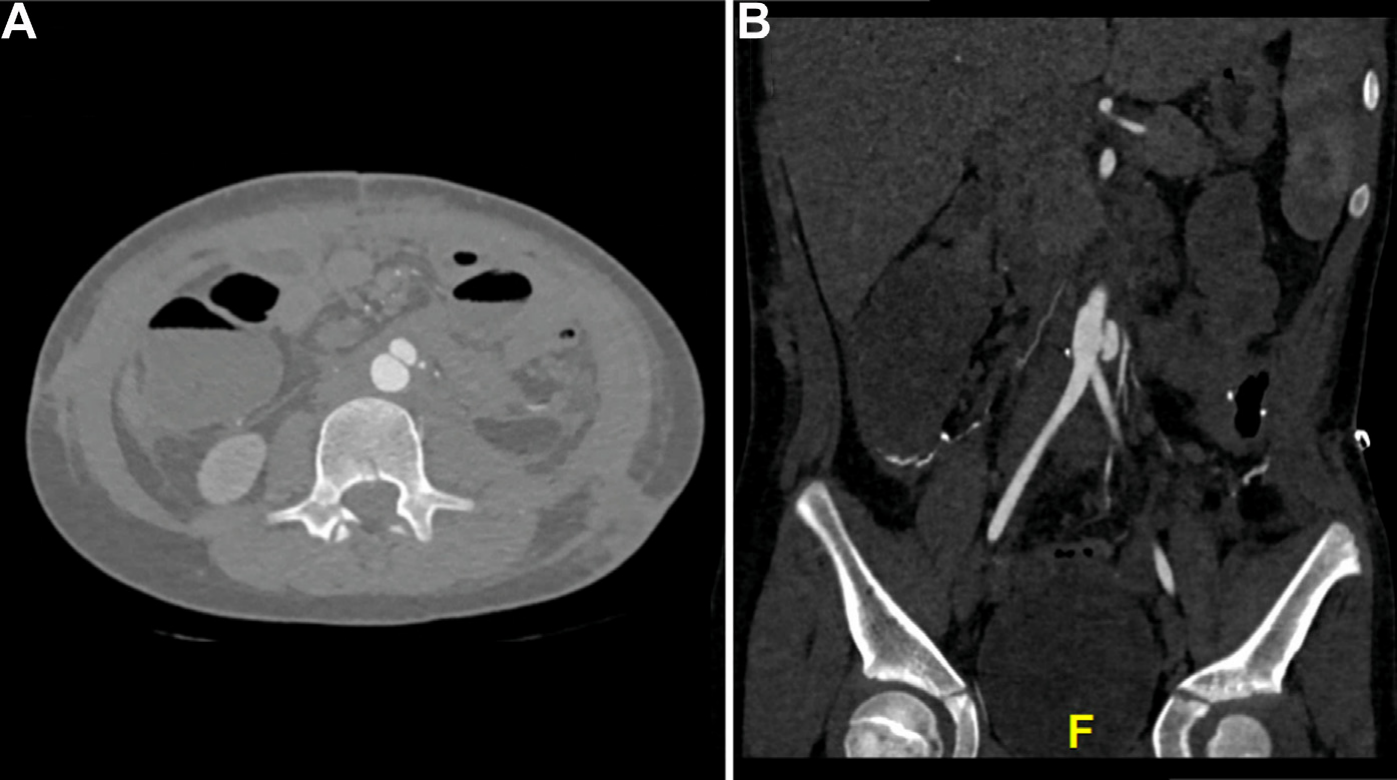
**A,** Computed tomography (CT) angiogram obtained 1 month after injury showing a rapidly degenerating aortic pseudoaneurysm. **B,** CT angiogram in coronal view with angle adjustment to demonstrate the location of the pseudoaneurysm in relation to the aortic bifurcation.

At 4 weeks after the intervention, the patient continued to have poor oral intake, total parenteral nutrition dependence, failure to thrive, and new-onset abdominal pain. She did not demonstrate any signs of infection, such as fevers or leukocytosis. A repeat CT scan was obtained, which demonstrated a pseudoaneurysm at the distal aorta near the aortic bifurcation (Fig 2). No radiographic signs suggestive of a mycotic aneurysm, such as arterial wall irregularity, surrounding stranding, or gas, were present. A multidisciplinary meeting was held to discuss potential treatment modalities. Significant concern was present regarding the need for any future open surgical treatment because the patient already had short gut syndrome with ∼100 cm of small bowel remaining and the likelihood of significant adhesions in the abdomen due to the extent of her abdominal injury and multiple rounds of surgery for bowel and abdominal wall reconstruction. It was evident that extensive adhesiolysis would be required to access the aorta, and the likelihood of possible enterotomy during the approach would be extremely high. Ultimately, the final decision was to proceed with an endovascular approach. The patient’s guardian provided written informed consent for the report of her case details and imaging studies.

## Description of technique

**Fig 3:**
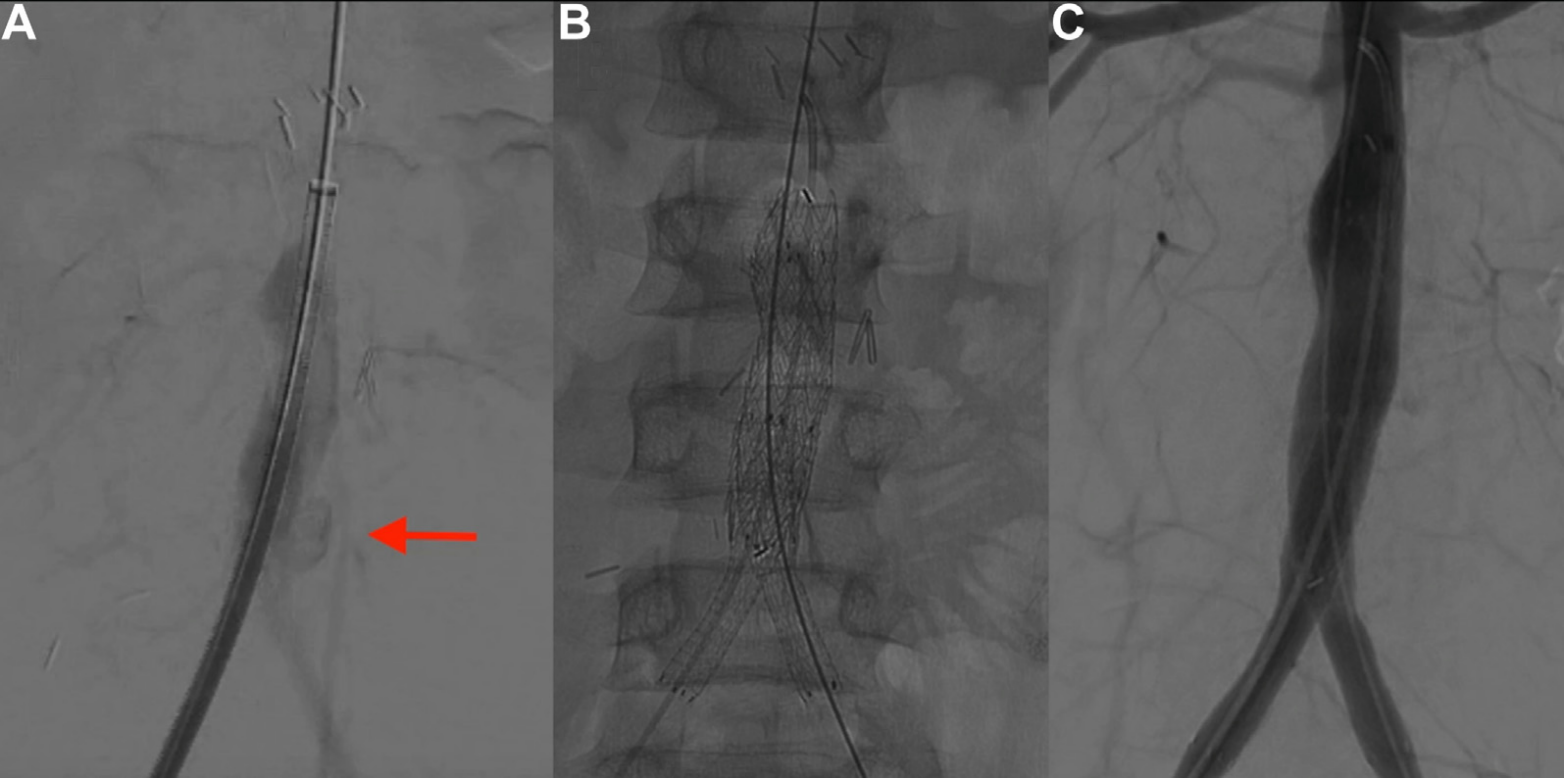
**A,** Initial angiogram showing the pseudoaneurysm at the aortic bifurcation. **B,** Intraoperative image of endovascular reconstruction of aortic bifurcation. **C,** Completion angiogram showing resolution of the aortic pseudoaneurysm without any evidence of an endoleak.

**Fig 4:**
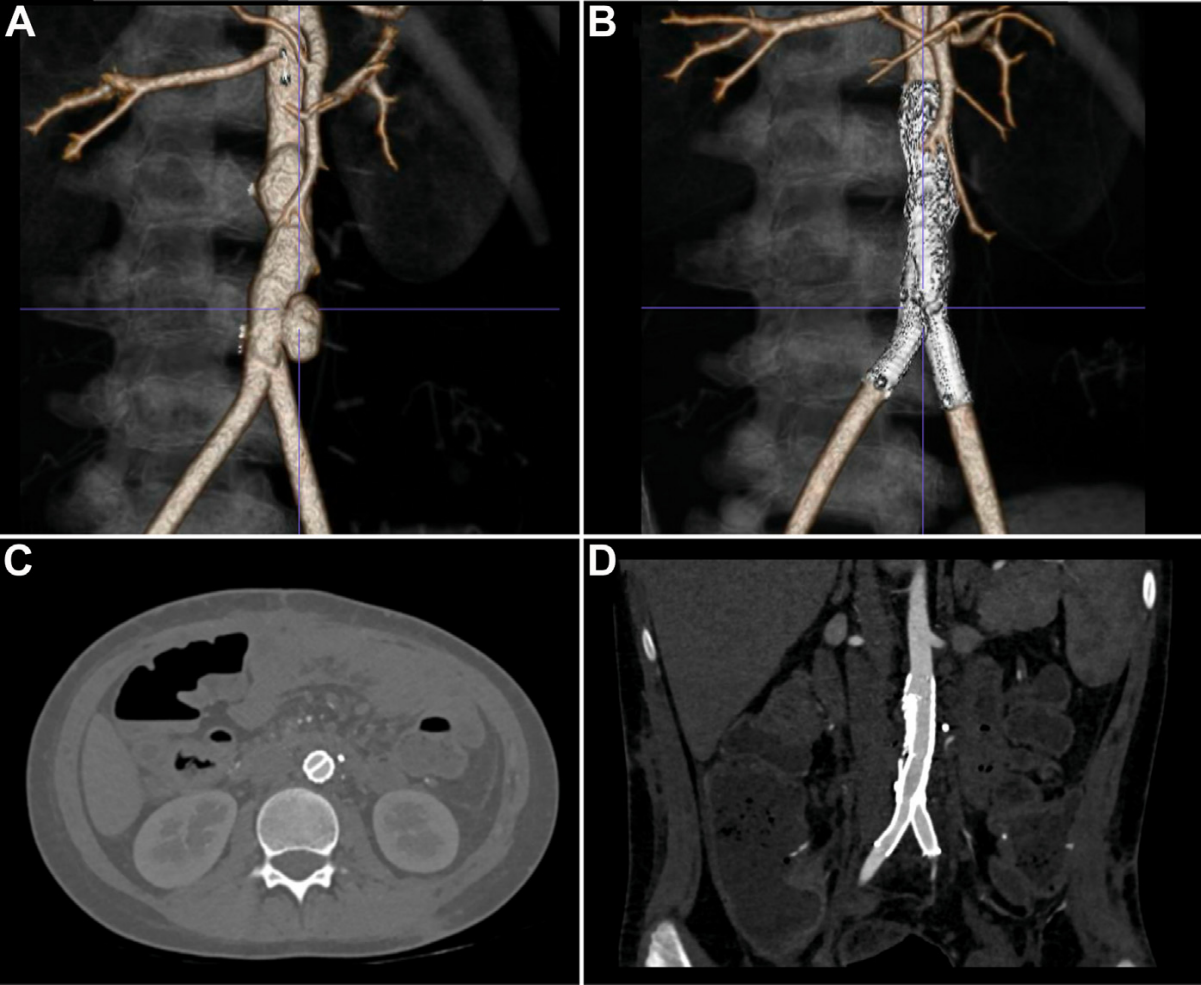
**A,** Three-dimensional reconstruction of computed tomography (CT) angiogram demonstrating the aortic pseudoaneurysm before intervention. **B,** Three-dimensional reconstruction of CT angiogram at the 6-month postoperative visit showing patent endografts with complete resolution of the pseudoaneurysm. **C,** Axial view of postoperative CT angiogram demonstrating complete obliteration of the gutters between the endografts. **D,** Coronal view of postoperative CT angiogram showing complete resolution of the pseudoaneurysm.

The CT angiogram was carefully reviewed for accurate endograft sizing. The infrarenal aorta measured 10.5 mm, and the distal aorta at the bifurcation measured 13.5 mm. The bilateral common iliac arteries measured 6 mm. Because none of the commercially aortic endografts would have fit the patient owing to the small aortic size, the limb of a Gore Excluder internal iliac limb endoprosthesis (W.L. Gore & Associates) was planned for use as the aortic endograft. Bilateral femoral cutdown for vascular access was performed, with a 12F and 8F sheath for the right and left femoral artery, respectively. Intravascular ultrasound and CT angiography of the abdominal aorta were used to size the grafts accurately and mark the exact location of the aortic bifurcation. The initial angiogram demonstrated the pseudoaneurysm near the aortic bifurcation (Fig 3, *A*). The Gore Excluder iliac limb endograft used for the aortic graft measured 16 mm × 14.5 mm × 7 cm. The iliac limb endograft was advanced from the right femoral artery access, with the 16-mm end positioned just above the aortic bifurcation and the 14-mm end placed in the smaller infrarenal aorta. The proximal extent of the endograft was placed several centimeters below the renal arteries. A hydrophilic guidewire and catheter were advanced through the left femoral access to cannulate the graft and gain access to the thoracic aorta. After confirmation of the wire’s location inside the aortic graft with intravascular ultrasound, the wire was exchanged for a 0.35-in. stiff wire. Next, bilateral, 8-mm, Viabahn kissing stents (W.L. Gore & Associates) were advanced, positioned crossing the bifurcation, and deployed at the same level. Next, two 10-mmViabahn stents were advanced proximal to the 8-mm stents and positioned up to the edge of the aortic cuff. The 10-mm Viabahn stents were deployed to raise the aortic bifurcation. Two 12-mm balloons were advanced up to the proximal edge of the Viabahn stents, and a kissing balloon technique was used to ensure expansion of the stent and obliterate the gutters (Fig 3, *B*). A completion angiogram was performed and showed the appropriate graft position with resolution of the pseudoaneurysm and bilateral renal artery preservation (Fig 3, *C*). The bilateral femoral artery accesses were closed with 6-0 Prolene interrupted sutures. The patient had an unremarkable postoperative course and was transferred back to the Children's Medical Center Dallas on postoperative day 3. The patient was discharged from the hospital on postoperative day 8. Her 6-month follow-up CT angiogram revealed widely patent endografts, with complete resolution of the pseudoaneurysm (Fig 4). The patient demonstrated appropriate recovery after surgery and was able to return to school and playing sports.

## Discussion

BAAT is an uncommon life-threatening condition in pediatric patients.^1^ Only 6% of blunt aortic injuries will occur in the abdomen compared with blunt thoracic aortic injury.^4^ It has been reported that more than one third of patients with BAAT who arrive at the hospital will die.^5^, ^6^, ^7^, ^8^ Consensus regarding the standard of care for BAAT has not yet been established. Focal intimal dissection or small contusions can be managed conservatively. However, close monitoring is necessary because it can progress to worsening injury, intraluminal thrombus, and/or pseudoaneurysm formation.^1^ Because of the high-energy impact causing these types of injuries, patients presenting with BAAT will often have associated intra-abdominal or spinal fractures requiring early intervention.^7^^,^^8^ This is especially true in cases of pediatric BAAT. Motor vehicle accidents are the most common mechanism of injury in the pediatric cohort. The injury occurs because the lap belt rides higher on pediatric patients over the abdomen rather than over the bony prominences of the pelvis, causing hyperflexion–distraction injuries and leading to multiple abdominal and vertebral injuries.^9^, ^10^, ^11^

Most reported cases of infrarenal aortic injuries have been managed with open repair. Replacement of an injured segment with a prosthetic graft is the most common form of repair. However, thromboendarterectomy with suturing of the intimal flap and extra-anatomic bypass are available options, depending on the degree of injury and stability of the patient.^5^ The number of reported studies on endovascular repair of infrarenal aortic injuries is limited.^3^^,^^12^^,^^13^ The reported cases of endovascular repair in pediatric patients are even rarer.^14^, ^15^, ^16^ Our presented case is very unique in that only one case, reported by Aidinian et al,^14^ has illustrated endovascular repair of the infrarenal aorta in a pediatric patient. To the best of our knowledge, the present study is the first reported case of bifurcated endovascular repair of BAAT. Endovascular repair of the bifurcation adds a significant challenge to the operator because no suitable aortic devices are commercially available that will fit the pediatric vasculature. Older pediatric patients in their mid- to late adolescence will likely be much better candidates for endovascular repair because their arteries will have reached their adult size. In our patient, the aortoiliac system was considerably smaller; thus, we elected to use the tapered iliac limb as the main body and reconstruct the iliac bifurcation using stents typically used in femoral arteries. The currently available endovascular therapies in the pediatric population have significant limitations. The calibers of the access vessels are significantly smaller; therefore, we have preferred to perform a cutdown and direct repair for vascular access instead of percutaneous techniques. Some have advocated alternative device delivery access, such as direct aortic cannulation.^15^ Long-term follow-up is imperative for pediatric patients undergoing endovascular stent graft repair. With our patient's growth, the potential exists for stent migration, stenosis, and/or occlusion. The long-term data on the performance of stent grafts in the pediatric population still needs to be elucidated. Any vascular intervention in a pediatric patient should be undertaken with anticipation of the patient's growth in height and an increase in the caliber of their vasculature. We performed our endograft repair as a last resort in this particularly challenging case. However, we anticipated that she will likely need further intervention in the future. Therefore, we ensured the presence of enough infrarenal aortic length to accommodate eventual endograft explantation and aortic replacement.

Several causes are possible for the early pseudoaneurysm formation after the initial open aortic repair. The leading hypothesis is incomplete debridement of the injured segment of the aorta leading to delayed pseudoaneurysm formation. Another possibility is the significant intra-abdominal contamination from devitalized intestines at the initial presentation, leading to suture line disruption. The integrity of the saphenous vein used was excellent. If the structural integrity of the saphenous vein was in question, it would have been more likely to present as a very late complication with aneurysmal degeneration of the entire patch. Pediatric blunt aortic injuries require close follow-up with surveillance with aortoiliac duplex ultrasound and the ankle brachial index. Cross-sectional imaging must be obtained if significant changes are found by noninvasive studies. Socioeconomic barriers such as moving to other states with more social and family support and the lack of insurance coverage for vascular service visits outside local pediatric health care institutions can result in loss to follow-up for ≤43% of patients.^15^^,^^17^

## Conclusions

BAAT is rare but associated with significant morbidity and mortality. In select cases, endovascular therapies can be used as a treatment option but will require meticulous preoperative planning and consideration of alternative vascular access and device use. Long-term follow-up is imperative because a number of patients could have delayed sequelae of the initial injury requiring intervention.
